# Genome-Wide Association Analysis of Pancreatic Beta-Cell Glucose Sensitivity

**DOI:** 10.1210/clinem/dgaa653

**Published:** 2020-09-18

**Authors:** Harshal A Deshmukh, Anne Lundager Madsen, Ana Viñuela, Christian Theil Have, Niels Grarup, Andrea Tura, Anubha Mahajan, Alison J Heggie, Robert W Koivula, Federico De Masi, Konstantinos K Tsirigos, Allan Linneberg, Thomas Drivsholm, Oluf Pedersen, Thorkild I A Sørensen, Arne Astrup, Anette A P Gjesing, Imre Pavo, Andrew R Wood, Hartmut Ruetten, Angus G Jones, Anitra D M Koopman, Henna Cederberg, Femke Rutters, Martin Ridderstrale, Markku Laakso, Mark I McCarthy, Tim M Frayling, Ele Ferrannini, Paul W Franks, Ewan R Pearson, Andrea Mari, Torben Hansen, Mark Walker

**Affiliations:** 1 Translational and Clinical Research Institute, Newcastle University, Newcastle upon Tyne, UK; 2 Novo Nordisk Foundation Center for Basic Metabolic Research, Faculty of Health and Medical Sciences, University of Copenhagen, Copenhagen, Denmark; 3 Department of Genetic Medicine and Development, University of Geneva Medical School, Geneva; 4 Institute of Neuroscience, National Research Council, Corso Stati Uniti 4, Padua, Italy; 5 Wellcome Centre for Human Genetics, Nuffield Department of Medicine, University of Oxford, Oxford, UK; 6 Oxford Centre for Diabetes, Endocrinology and Metabolism, Radcliffe Department of Medicine, University of Oxford, Oxford, UK; 7 Department of Clinical Sciences, Genetic and Molecular Epidemiology Unit, Skåne University Hospital Malmö, Lund University, 205 02 Malmö, Sweden; 8 Integrative Systems Biology Group, Department of Health Technology, Technical University of Denmark (DTU), Kemitorvet, Building 208, 2800 Kgs. Lyngby, Denmark; 9 Center for Clinical Research and Disease Prevention, Bispebjerg and Frederiksberg Hospital, The Capital Region, Copenhagen, Denmark; 10 Department of Clinical Medicine, Faculty of Health and Medical Sciences, University of Copenhagen, Copenhagen, Denmark; 11 Section of General Practice, Institute of Public Health, Faculty of Health Sciences, University of Copenhagen, Øster Farimagsgade 5, Copenhagen, Denmark; 12 Novo Nordisk Foundation Centre for Basic Metabolic Research (Section of Metabolic Genetics), Faculty of Health and Medical Sciences, University of Copenhagen, Copenhagen, Denmark; 13 Department of Public Health (Section of Epidemiology), Faculty of Health and Medical Sciences, University of Copenhagen, Copenhagen, Denmark; 14 Department of Nutrition, Exercise and Sports (NEXS), Faculty of Science, University of Copenhagen, Copenhagen, Denmark; 15 Eli Lilly Regional Operations Ges.m.b.H., Koelblgasse 8–10, Vienna, Austria; 16 Genetics of Complex Traits, University of Exeter Medical School, University of Exeter, Exeter, UK; 17 Diabetes Division, Sanofi-Aventis Deutschland GmbH, Frankfurt, 65926 Frankfurt am Main, Germany; 18 NIHR Exeter Clinical Research Facility, University of Exeter Medical School, Exeter, UK; 19 Department of Epidemiology and Biostatistics, VUMC, de Boelelaan 1089a, HV, Amsterdam, the Netherlands; 20 Department of Endocrinology, Abdominal Centre, Helsinki University Hospital, Helsinki, Finland; 21 Department of Clinical Sciences, Diabetes & Endocrinology Unit, Lund University, Skåne University Hospital Malmö, CRC, 91-12, 205 02, Malmö, Sweden; 22 Institute of Clinical Medicine, Internal Medicine, University of Eastern Finland, Kuopio, Finland; 23 Oxford Centre for Diabetes Endocrinology and Metabolism, University of Oxford, Oxford, UK; 24 CNR Institute of Clinical Physiology, Pisa, Italy; 25 Department of Nutrition, Harvard TH Chan School of Public Health, Boston, Massachusetts; 26 Department of Public Health and Clinical Medicine, Umeå University, Umeå, Sweden; 27 Division of Population Health & Genomics, School of Medicine, University of Dundee, Dundee, UK

**Keywords:** Glucose intolerance, diabetes progression, beta-cell function, incretin, mathematical model

## Abstract

**Context:**

Pancreatic beta-cell glucose sensitivity is the slope of the plasma glucose-insulin secretion relationship and is a key predictor of deteriorating glucose tolerance and development of type 2 diabetes. However, there are no large-scale studies looking at the genetic determinants of beta-cell glucose sensitivity.

**Objective:**

To understand the genetic determinants of pancreatic beta-cell glucose sensitivity using genome-wide meta-analysis and candidate gene studies.

**Design:**

We performed a genome-wide meta-analysis for beta-cell glucose sensitivity in subjects with type 2 diabetes and nondiabetic subjects from 6 independent cohorts (n = 5706). Beta-cell glucose sensitivity was calculated from mixed meal and oral glucose tolerance tests, and its associations between known glycemia-related single nucleotide polymorphisms (SNPs) and genome-wide association study (GWAS) SNPs were estimated using linear regression models.

**Results:**

Beta-cell glucose sensitivity was moderately heritable (*h*^*2*^ ranged from 34% to 55%) using SNP and family-based analyses. GWAS meta-analysis identified multiple correlated SNPs in the *CDKAL1* gene and *GIPR-QPCTL* gene loci that reached genome-wide significance, with SNP rs2238691 in *GIPR-QPCTL* (*P* value = 2.64 × 10^−9^) and rs9368219 in the *CDKAL1* (*P* value = 3.15 × 10^−9^) showing the strongest association with beta-cell glucose sensitivity. These loci surpassed genome-wide significance when the GWAS meta-analysis was repeated after exclusion of the diabetic subjects. After correction for multiple testing, glycemia-associated SNPs in or near the *HHEX* and *IGF2B2* loci were also associated with beta-cell glucose sensitivity.

**Conclusion:**

We show that, variation at the *GIPR-QPCTL* and *CDKAL1* loci are key determinants of pancreatic beta-cell glucose sensitivity.

Decreased insulin secretion secondary to impaired pancreatic beta-cell function is an essential element in the development of abnormal glucose tolerance and type 2 diabetes. Using a progressive, stepped intravenous glucose infusion, a dose-response curve can be generated for insulin secretion rates against plasma glucose levels. In cross-sectional studies, the slope of this curve (termed beta-cell glucose sensitivity) progressively decreases from normal to impaired glucose tolerance, and through to type 2 diabetes (1). An analogous dose-response relationship can be derived from standard oral glucose and mixed meal tolerance tests (OGTT and MMTT, respectively) using C-peptide kinetic analysis to measure insulin secretion rates (2). This approach offers several advantages. First, it assesses beta-cell glucose sensitivity under conditions that reflect daily living in contrast to intravenous glucose–based methods that exclude the incretin system. Second, it is independent of potential confounders, such as hepatic insulin clearance, that can influence circulating insulin levels and impact on measures of beta-cell function that examine changes in insulin levels in response to a glucose challenge.

In line with the studies using intravenous glucose infusion, we have shown that the model-based beta-cell glucose sensitivity decreases with progressive glucose intolerance using cross-sectional data (3). Crucially, beta-cell glucose sensitivity was a strong, independent predictor of deteriorating glucose tolerance (4) and the development of type 2 diabetes (5) in longitudinal follow-up studies of people free from diabetes. Furthermore, beta-cell glucose sensitivity, together with a model-derived measure of whole-body insulin sensitivity, was found to completely replace the classical clinical risk factors (such as obesity and plasma glucose concentrations) as predictors of deteriorating glucose tolerance (4). In view of the emerging importance of beta-cell glucose sensitivity as a predictor of deteriorating glucose tolerance, we conducted a genome-wide analysis to understand the genetic basis of this phenotype.

The aims of this study were to define the heritability of beta-cell glucose sensitivity and to perform genome-wide association and candidate gene (known diabetes and glycemic risk loci) association analyses for beta-cell glucose sensitivity across a range of glucose tolerance.

## Methods

### Cohort description

The discovery cohorts were 2 multicenter prospective cohort studies within the Innovative Medicines Initiative Diabetes Research on Patient Stratification (IMI DIRECT) Consortium (6), which were specifically designed to address the molecular basis to glycemic deterioration. The IMI DIRECT cohorts include detailed information and biomaterials suitable for the analysis of genetic and nongenetic biomarkers for glycemic deterioration before and after the onset of type 2 diabetes. Cohort 2.1 (n = 2233) enrolled people with normal and dysregulated, but not diabetic, glucose homeostasis based on HbA1c (5.7%–6.4%, 40–48 mmol/mol) and OGTT, while cohort 2.2 consisted of those (n = 784) who had been recently diagnosed with type 2 diabetes at the time of enrollment. The study design and sample selection are previously described (7).

The data for the replication analyses (which has been meta-analyzed with the discovery cohorts) came from 4 independent cohorts, consisting of a mix of volunteers spanning a range from normal glucose tolerance to type 2 diabetes. These were the Relationship between Insulin Sensitivity and Cardiovascular disease (RISC) study (8), the ADIGEN study (9) the 1936-cohort (10) and the Family study (11). The RISC study is a prospective study of 1276 men and women with normal glucose tolerance of European ancestry, aged from 30 to 60 years, from 20 centers in 13 European countries (8). The ADIGEN study was a follow-up examination at around the age of 50 years of 2 groups of young men assessed for military service at around 19 years of age between 1943 and 1977 in the metropolitan area of Copenhagen (Denmark); 1 group were the most obese in that population (n = 248) and the control group (n = 320) was a random selection of 0.5% of that population. The study was designed to investigate frequent functional genetic variants that influence the development of obesity. The 1936*-*cohort is a population-based prospective age-specific cohort that consists of 1198 Danish subjects born in 1936, who were resident in municipalities nearby Glostrup Hospital (Denmark) in 1976. In the present study, we included subjects participating in the 20-year follow-up in 1996 (12). The purpose of the study was to follow and examine the association between insulin sensitivity and the development of cardiovascular diseases. The Family study consists of approximately 95 families from the Copenhagen area, including a total of 533 individuals, of whom 336 individuals were included in the present study. Families were recruited if one parent had type 2 diabetes. The study was designed to identify genetic loci influencing glucose homeostasis using linkage methods in families with type 2 diabetes. OGTT and MMTT were conducted as previously described (7-11). Briefly, following an overnight fast, blood was sampled at baseline (0 minutes) and at 30-minute intervals for 2 hours following the oral glucose/meal challenge. Blood was assayed for plasma glucose, insulin, and C-peptide at a central quality control laboratory for each cohort.

### Glucose sensitivity measurement

Beta-cell function was assessed from the OGTT and MMTT (see Table 1) using a model that describes the relationship between insulin secretion and glucose concentration, which has been described in detail previously (2, 3). Glucose sensitivity measures were determined from the baseline OGTT and MMTT data for each cohort. Glucose sensitivity is the mean slope over the observed glucose range of the model-determined dose-response that relates insulin secretion to glucose concentration during the OGTT or MMTT. As shown in previous studies (13), glucose sensitivity reflects both intrinsic beta-cell function, as tested by intravenous glucose infusion, and the effects of incretin hormones. All analyses were conducted by 3 operators supervised by A. Mari.

**Table 1.** Demographic Characteristics and Key Metabolic Parameters of the Study Population for the GWAS Meta-analysis of Beta-Cell Glucose Sensitivity (n = 5706)

	IMI DIRECT 2.1	IMI DIRECT 2.2	RISC study	1936 birth cohort	ADIGEN study	Family study
	(n = 2233)	(n = 784)	(n = 1276)	(n = 622)	(n = 455)	(n = 336)
Age, years, mean (±SD)	62.2 (±6.50)	61.9 (±7.91)	44.06 (±8.34)	60.5 (±0.46)	48.9 (±5.81)	44.7 (±13.3)
Ethnicity	Caucasian	Caucasian	Caucasian	Caucasian	Caucasian	Caucasian
Women, %	24%	43%	54%	53%	0%	56%
% with diabetes	0%	100%	0%	6%	10%	9.5%
BMI, mean (±SD)	28.1(±4.01)	30.49 (±4.99)	25.68 (±4.13)	26.7 (±3.99)	30.2 (±6.78)	26.8 (±4.90)
Oral challenge	OGTT	MMTT	OGTT	OGTT	OGTT	OGTT
Glucose sensitivity, pmol min^-1^ m^-2^ mmol/L^-1^, (median±SD)	97.7 (±1.6)	69.1 (±1.8)	107.1 (±1.8)	85.1 (±66)	69.1 (±43.6)	61.6 (±40.7)
Platform	Illumina HumanCore array	Illumina HumanCore array	Affymetrix	llumina HumanCore Exome-24 BeadChip	Human610-Quad v.1.0 BeadChip	Illumina HumanCore Exome-24 BeadChip
pHWE exclusion	<0.0001	<0.0001	<0.0001	<0.0001	<0.0001	<0.0001
Imputation software	IMPUTE	IMPUTE	IMPUTE	IMPUTE	IMPUTE	IMPUTE
GWAS software	SNPTEST	SNPTEST	SNPTEST	SNPTEST	SNPTEST	Mixed model using GCTA version 1.91.2
NCBI Build for imputation	GRCh38	GRCh38	GRCh38	GRCh38	GRCh38	GRCh38

Abbreviations: BMI, body mass index; pHWE, *P* value for Hardy-Weinberg equilibrium; IMI DIRECT, Innovative Medicines Initiative Diabetes Research on Patient Stratification; RISC, Relationship between Insulin Sensitivity and Cardiovascular disease study.

### Genotyping and imputation methods

Pre-imputation quality control was standardized across all the 6 cohorts with minor allele frequency cutoff of 0.01, and sample and single nucleotide polymorphism (SNP) call rate of >0.98. Genotype imputation involved a 2-step process: (i) the genotypes to be imputed were “pre-phased” (a statistical method is applied to genotype data to infer the underlying haplotypes of each individual) using SHAPEIT (14); and (ii) IMPUTE (15) was then used to combine the inferred haplotypes with a reference panel of haplotypes and impute the unobserved genotypes in each sample using the 1000 Genomes Phase 3 (October 2014 release). Imputation was carried out in chunks of 1 Mb with a 500-kb buffer region. Imputed variants in each nonoverlapping part of each chunk were concatenated into per chromosome files.

### Statistical analysis

#### Heritability estimation.

Narrow-sense heritability for glucose sensitivity was estimated using the GCTA software (16) and the directly genotyped markers from the 2 IMI DIRECT cohorts. We then estimated univariate heritability of glucose sensitivity by the restricted maximum likelihood method in GCTA (with sex and age at baseline included as covariates). The heritability thus estimated is also known as “narrow-sense” or “chip” heritability which is an indicator of additive genetic contribution from all SNPs. We estimated the heritability of beta-cell glucose sensitivity in the Family study using the tool SOLAR (17). We used 2 different models to estimate the heritability of the trait in the Family study. The first model includes the additive genetic influence and the unique environment (AE model). The second model uses the additive genetic influence, the shared environment (household effect), and the unique environment (ACE model).

#### Candidate gene selection and analysis.

For candidate gene analysis, we selected 155 SNPs associated with type 2 diabetes and glycemic and insulin-related traits reported in previous studies (18, 19). This SNP set was used by a recent GWAS of first-phase insulin secretion, measured by intravenous glucose tolerance tests (20). Linear regression models adjusted for age, sex, body mass index (BMI), first 3 principal components of ancestry, and study center were used to test the association of each SNP with glucose sensitivity. For this analysis, a *P* value ≤ 0.0003 (0.05/number of tests) was considered statistically significant.

#### Genome-wide association analysis.

We performed genome-wide association study (GWAS) of beta-cell glucose sensitivity in 5706 individuals of European descent. In the primary analyses, glucose sensitivity measures were fitted in a linear regression model with age, gender, and study center (for RISC and DIRECT studies, which were conducted at multiple centers), BMI, and the first 3 principal components for race/ethnicity (derived from EIGENSTRAT) included as covariates. We also ran the analysis without adjustment for BMI in an attempt to identify loci associated with glucose sensitivity via adiposity. The glucose sensitivity was normalized by log10 transformation. To account for imputation uncertainty, we used the 1000 Genomes imputation allele dosage in linear models.

#### Meta-analysis of genome-wide association studies.

We used the METAL program (21) to meta-analyze individual studies by combining the study-specific *P* values across studies taking sample size and direction of effect into account. In total, 8 978 282 SNPs passed quality control (minor allele frequency [MAF] 2% in individual cohorts; imputation quality >0.3 in MACH or >0.4 in IMPUTE) and were included in the meta-analysis. METAL was also used to assess heterogeneity across the 3 cohorts for the top signals.

#### eQTL analyses.

To identify potential effector transcripts mediating the activity of the top associated variants, we extracted cis-expression quantitative trait loci (eQTLs) information available from each of the top associated SNPs in 43 GTEx tissues (22) and pancreatic islets (23). Since these studies reported multiple genes associated with each SNP, we selected the most strongly associated eQTLs per SNP that generated a nominal *P* value, before calculating a corrected *P* value with the p.adjust function in R and using the Bejamini-Hochberg method.

## Results

### Demographic and key metabolic characteristics of the study population

Table 1 summarizes the demographic characteristics of the study population (n = 5706). All the participating cohorts comprised individuals of both genders, except the ADIGEN study which recruited just males.

### Heritability estimates for beta-cell glucose sensitivity

The SNP-based heritability (narrow-sense heritability) of beta-cell glucose sensitivity in the combined discovery cohort, type 2 diabetes, and prediabetes population (n = 3017) after adjustments for age, sex, and BMI was 34% (*h*^*2*^ = 0.34 [±0.09] *P* value = 2.33 × 10^−10^).

In the Family study, after adjustments for age, sex, and BMI and using the inverse transformed phenotype in an AE model, we obtained a heritability of 55% (standard error [SE] 13%; *P* value = 7.12 × 10^−9^). Analysis with the ACE model did not change the result, with zero shared environmental effect (C). The heritability was also calculated using only people with normal glucose tolerance (n = 252) with the same parameters. Both the AE model and the ACE model (with zero variance explained by shared environment) gave a heritability of 52% (SE 12%; *P* value = 2.73 × 10^−8^). Thus, the heritability did not seem to be affected by altered glucose tolerance.

### Genome-wide association study and meta-analysis

**Figure 1.**
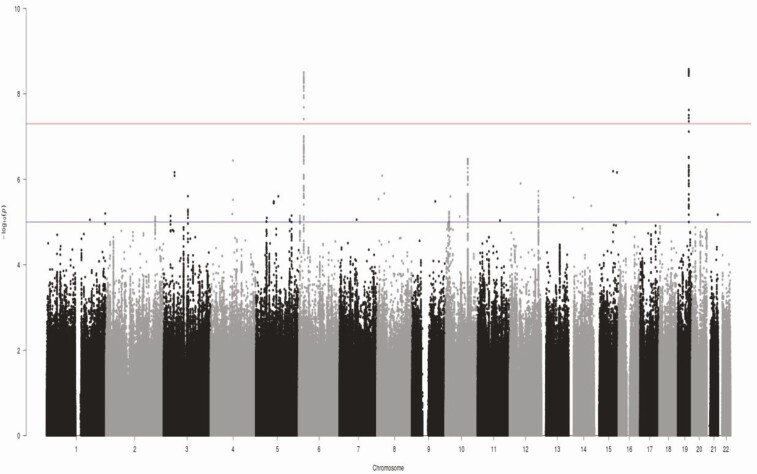
Manhattan plot of genome-wide *P* values of association for beta-cell glucose sensitivity: horizontal upper and lower lines represent the suggestive genome-wide significance thresholds of p <10^−7^ and p <10^–5^, respectively.

**Figure 2.**
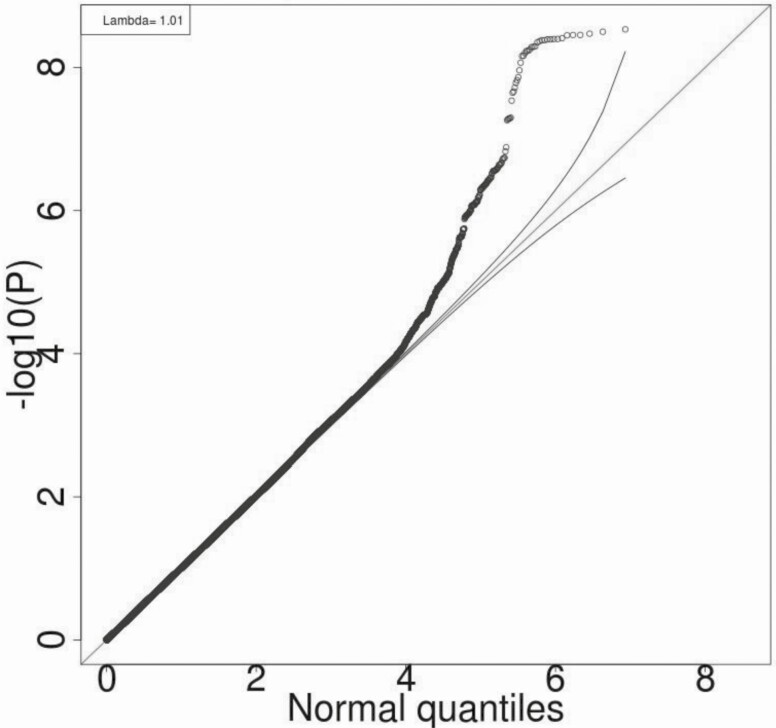
Q-Q plot of genome-wide *P* values of association for beta-cell glucose sensitivity of the observed versus expected *P* values given the number of statistical tests performed for beta-cell glucose sensitivity.

**Figure 3.**
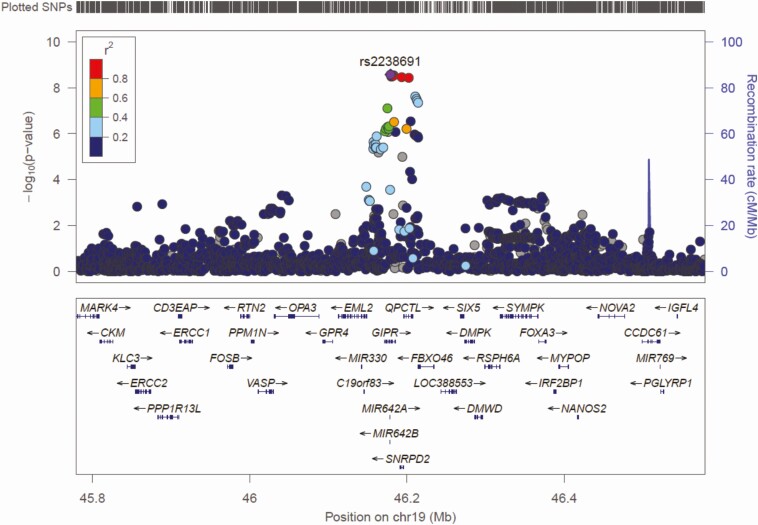
Regional association plot of *GIPR-QPCTL* gene region. Plot produced in Locus Zoom with the most strongly associated SNP (rs2238691) shown as the purple diamond.

**Figure 4.**
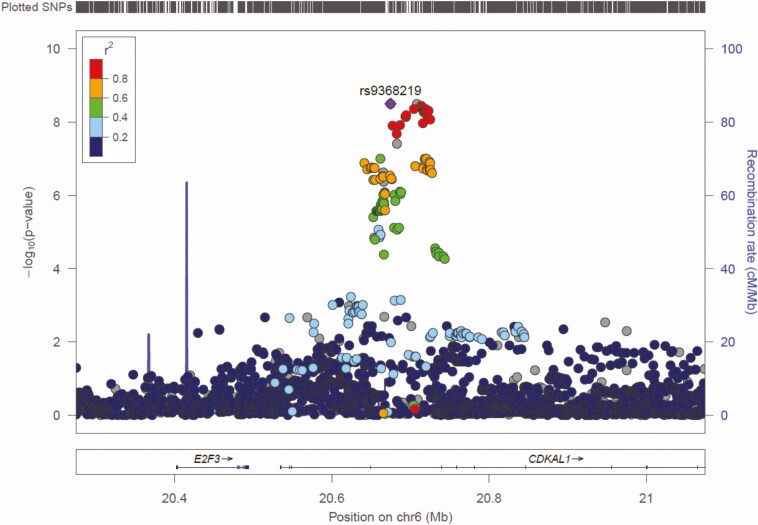
Regional association plot of *CDKAL1* gene region. Plot produced in LocusZoom with the most strongly associated SNP (rs9368219) shown as the purple diamond.

Figs. 1 and 2 show the Manhattan and Q-Q plots, respectively, for the GWAS meta-analysis across the 6 cohorts. The Q-Q plots for individual cohorts are shown in Supplementary Figure 1 (6). The GWAS meta-analysis showed multiple correlated SNPs on chromosome 6 in the *CDKAL1* gene locus and on chromosome 19 in the *GIPR-QPCTL* gene region reaching the accepted level of significance for GWAS with *P* value < 10^–8^ (all significant SNPs listed in Supplementary Table 1 (6)). Greatest significance was seen for SNP rs2238691 (Z-score = −5.953, *P* value = 2.64 × 10^−9^) within the *GIPR-QPCTL* region and for rs9368219 (Z-score −5.9, *P* value = 3.15 × 10^−9^) in the *CDKAL1* region (Figs 3 and 4, respectively). The effect estimates for these SNP were comparable across the replication and discovery cohorts. The SNPs with a *P* value between >10^–8^ and <10^–7^ are summarized in Supplementary Table 2 (6).

To further explore the top associated variants, we extracted cis-expression quantitative trait loci (cis-eQTLs) associations from 43 GTEx tissues (22) and pancreatic islets from the InsPIRE study (23). Cis-eQTLs analysis is used to explore candidate genes mediating the activity of GWAS variants. By extracting the most significant eQTLs per tissue for each of the SNPs included in Supplementary Table 1 (6), we found that the most strongly associated eQTLs were all expressed in pancreatic islets (Supplementary Table 3 (6)), although no individual eQTL was significant after correction for multiple testing.

In view of the potential secondary metabolic effects of the diabetic state on pancreatic beta-cell function, the GWAS meta-analysis was repeated in nondiabetic subjects (n = 4544) after excluding the IMI DIRECT 2.2 cohort and the known diabetic patients in the other cohorts listed in Table 1. There was no change in the SNPs that achieved genome-wide significance (Supplementary Table 4 (6)), although the top SNPs identified in each of the *CDKAL1* and *GIPR-QPCTL* regions were different (rs1040558 and rs35541137, respectively).

### Candidate gene association tests

Table 2 shows the association of known glycated hemoglobin A1c (HbA1c), glycemic traits (fasting and 2-hour plasma glucose) and type 2 diabetes SNPs with beta-cell glucose sensitivity (*P* < 0.01). After correction for multiple testing, type 2 diabetes–associated SNPs in or near *HHEX, CDKAL1, IGF2B2*, fasting glucose–associated SNPs in or near *CDKAL1* and *IGF2BP2*, and a 2-hour glucose post-OGTT–associated SNP at the *GIPR* locus were all associated with beta-cell glucose sensitivity. The association of these SNPs was directionally consistent with the expected underlying biology. For instance, the “T” allele at rs1111875 in the *HHEX locus* is protective for diabetes and is associated with higher beta-cell glucose sensitivity. This SNP was nominally associated with the expression of the *MARK2P9* gene in pancreatic islets (*P* value = 6.35 × 10^−3^), but not with eQTLs in other tissues (Supplementary Table 5 (6)). Other variants included in the analysis were also significant cis-eQTLs in pancreatic islets, this being the only tissue with significant eQTLs after multiple testing for the candidate SNPs listed in Table 2. The associations of the 155 known loci for type 2 diabetes HbA1c and other glycemic traits with beta-cell glucose sensitivity are summarized in Supplementary Table 6 (6). The heterogeneity in the effect sizes for the top SNPs in Table 2 across the cohorts was not significant (Supplementary Table 7 (6)).

**Table 2.** Association of the Known SNPs for Type 2 Diabetes, HbA1c, and Glycemic Traits With Beta-Cell Glucose Sensitivity

Phenotype	CHR	SNP	Position	Effect allele	Z-score	*P* value	Gene
2-hr glucose	19	rs11672660	46180184	t	-5.92	3.21E-09	*GIPR*
Fasting glucose	6	rs9368222	20686996	a	-4.907	9.25E-07	*CDKAL1*
T2D	6	rs7756992	20679709	a	4.902	9.47E-07	*CDKAL1*
T2D	10	rs1111875	94462882	t	4.661	3.15E-06	*HHEX*
Fasting glucose	3	rs7651090	185513392	a	3.753	1.75E-04	*IGF2BP2*
T2D	3	rs4402960	185511687	t	-3.751	1.76E-04	*IGF2BP2*
FGFproinsulin	11	rs11603334	72432985	a	3.366	7.62E-04	*ARAP1*
T2D	11	rs1552224	72433098	a	-3.366	7.63E-04	*ARAP1*
Fasting glucose	13	rs11619319	28487599	a	3.16	1.58E-03	*PDX1*
Fasting glucose	2	rs560887	169763148	t	-3.097	1.95E-03	*G6PC2*
T2D	7	rs849135	28196413	a	2.926	3.43E-03	*JAZF1*
HbA1c	2	rs552976	169791438	a	-2.902	3.71E-03	*ABCB11*
Fasting glucose	7	rs6943153	50791579	t	2.85	4.37E-03	*GRB10*
T2D	19	rs8108269	46158513	t	2.794	5.20E-03	*GIPR*
Fasting glucose	11	rs174550	61571478	t	-2.769	5.63E-03	*FADS1*
Fasting glucose	11	rs174576	61603510	a	2.607	9.15E-03	*FADS1*

Phenotype refers to the phenotype reported to be associated with this SNP.

Associations reaching Bonferroni equivalents of *P* < 0.05 are in bold. Base pair position build-37.p13.

Abbreviations: CHR, chromosome; HbA1c, glycated hemoglobin A1c; SNP, single nucleotide polymorphism; T2D, type 2 diabetes.

The same candidate gene analyses were repeated in just the nondiabetic subjects. The associations between SNPs and beta-cell glucose sensitivity observed in the whole cohort (Table 2) remained in the nondiabetic subjects (Supplementary Table 8 (6)), albeit at generally lesser degrees of statistical significance.

For the known SNPs associated with type 2 diabetes and glycemic traits (*P* < 0.01), we examined the overlap between their association with beta-cell glucose sensitivity in our study and the early peak insulin response to an intravenous glucose challenge (IVGTT) as previously reported (19). As shown in Supplementary Figure 2 (6), SNPs in *GIPR, G6PC2, JAZF1, and FADS1* are associated with pancreatic beta-cell glucose sensitivity but not with early insulin response during the IVGTT, while SNPs in *ABCB11, CDKAL1, IGF2BP2, ARAP1, HHEX, PDX1*, and *GRB10* show an association with both phenotypes. Conversely, variants in *MNTR1B* and *TCF7L2* were strongly associated with the early insulin response during the IVGTT, but variation in these type 2 diabetes susceptibility loci was not associated with pancreatic beta-cell glucose sensitivity after correction for multiple testing.

## Discussion

This is the first study to conduct a genome-wide association meta-analysis of pancreatic beta-cell glucose sensitivity. The key finding is that variation at the *CDKAL1* and *GIPR-QPCTL* regions showed the strongest associations with beta-cell glucose sensitivity in the entire study cohort, and when the analysis was limited to nondiabetic subjects. These findings were corroborated by the candidate gene analyses, which also found a strong association between known variants at the *HHEX* locus and beta-cell glucose sensitivity. In addition, we observed that pancreatic islet eQTLs clustered with the top GWAS SNPs, while multiple variants from the candidate gene SNPs were individually associated with islet eQTLs.

We have previously shown that type 2 diabetes risk variants in *CDKAL1* and *HHEX* were associated with decreased beta-cell glucose sensitivity in nondiabetic individuals (24); however, these associations have not been tested in a larger cohort that includes subjects with abnormal glucose tolerance. Lyssenko and colleagues reported similar findings in a large longitudinal cohort study but measured the acute insulin response to an oral glucose load rather than beta-cell glucose sensitivity (25).

*CDKAL1* represents one of the strongest signals of association with type 2 diabetes across diverse ancestries, with minimal heterogeneity in allelic effects between populations (26-30). The role of *CDKAL1* in pancreatic beta-cell function remains to be fully defined. However, it is strongly expressed in human adult pancreatic islets relative to other tissues (31), and *CDKAL1* gene deletion is accompanied by modestly impaired insulin secretion during high-fat feeding in mice (28). There is emerging evidence that *CDKAL1* encodes a methylthiotransferase that regulates tRNA^Lys^ function and proinsulin synthesis in pancreatic beta cells (32).

A large GWAS (33) identified the *GIPR-QPCTL* (rs10423928) locus to be associated with 2-hour blood glucose levels after an oral glucose challenge. This study also showed that *GIPR* had strong specific mRNA expression in the sorted pancreatic beta cells, supporting the role of *GIPR* in insulin secretion. The *GIPR-QPCTL* (rs10423928) locus is in linkage disequilibrium with rs2238691 identified in our study (r^2^ = 41% in HapMap CEU Population and *r*^*2*^ = 99% in our study) suggesting that these 2 GWAS identified the same signal. Gastric inhibitory polypeptide (GIP) along with glucagon-like peptide-1 (GLP-1) are incretin hormones that serve to amplify the insulin secretory response after food ingestion, and *GIPR* plays a key role in this process. As previously reported, the beta-cell glucose sensitivity is in part influenced by the effects of incretin hormones (13). Interestingly, variation at the GLP-1 receptor gene locus was not associated with beta-cell glucose sensitivity in this analysis.

A recent study reported a similar approach to investigate the genetic basis of the early insulin response to an IVGTT (20). The strongest associations were in or near the *MTNR1B* and *CDKAL1* loci. Taken with our findings, the evidence highlights a critical role for *CDKAL1* in the regulation of pancreatic insulin secretion (Supplementary Figure 2 (6)). Intriguingly, although variation in *TCF7L2* has been identified as the strongest common genetic determinant of type 2 diabetes, it was not a significant determinant of beta-cell glucose sensitivity but was associated with the early insulin response to an IVGTT (20).

We show that the known loci for HbA1c, such as *ABCB11*, and the established type 2 diabetes loci*, IGF2BP2, and ARAP1*, could mediate their effect on type 2 diabetes risk by their action on pancreatic beta-cell glucose sensitivity. Previous literature has shown the association of these loci with glucose homeostasis and cis-eQTLs active in pancreatic islets (23). Insulin-like growth factor 2 (IGF2) mRNA-binding protein 2 (*IGF2BP2*) belongs to a family of IGF2 mRNA-binding proteins that play an important role in pancreatic development (34), while *IGF2BP2* mRNA levels are associated with glucose and insulin homeostasis (35).

A limitation of our study is its modest sample size of 5706 European samples for a GWAS study, which constrains our ability to detect associations with low-frequency variants. Another potential concern is that beta-cell glucose sensitivity was determined from OGTT and MMTT. However, the MMTT was used only in the DIRECT 2.2 cohort and GWAS analyses were first conducted separately within the individual cohorts, and then the cohort specific *P* values were meta-analyzed. Furthermore, when the DIRECT 2.2 cohort was excluded as part of the analysis of the nondiabetic subjects, variation in *CDKAL1* and *GIPR-QPCTL* regions remained the strongest determinants of beta-cell glucose sensitivity.

Clearly, the measurement of pancreatic beta-cell glucose sensitivity as a predictor of type 2 diabetes would be impractical in the clinical setting. The identification, therefore, of clinically applicable biomarkers of beta-cell glucose sensitivity is attractive, and as a step toward this goal we have explored the genetic architecture of this pancreatic beta-cell phenotype.

In summary, *CDKAL1* and *GIPR-QPCTL* loci showed the strongest associations with beta-cell glucose sensitivity by genome-wide and candidate gene-based approaches, and these associations were independent of diabetes status.

## Data Availability

The datasets generated during and/or analyzed during the current study are not publicly available but are available from the corresponding author on reasonable request.

## Abbreviations

ACE model: model using additive genetic influence, shared environment (household effect), and the unique environment
AE model: model using additive genetic influence and the unique environment
BMI: body mass index
eQTL: expression quantitative trait loci
GWAS: genome-wide association study
HbA1c: glycated hemoglobin A1c
IGF2: insulin-like growth factor 2
IMI DIRECT: Innovative Medicines Initiative Diabetes Research on Patient Stratification
IVGTT: intravenous glucose challenge
MAF: minor allele frequency
MMTT: mixed meal tolerance test
OGTT: oral glucose tolerance test
RISC: Relationship between Insulin Sensitivity and Cardiovascular disease study
SE: standard error
SNP: single nucleotide polymorphism

## Additional Information

***Disclosure Summary:*** The authors declare that there are no conflicts of interest related to this manuscript.
