# Corrigendum to: “Genome-Wide Association Analysis of Pancreatic Beta-Cell Glucose Sensitivity”

**DOI:** 10.1210/clinem/dgaa725

**Published:** 2020-10-11

In the above-named article by Deshmukh HA, Madsen AL, Viñuela A, Have CT, Grarup N, Tura A, Mahajan A, Heggie AJ, Koivula RW, De Masi F, Tsirigos KK, Linneberg A, Drivsholm T, Pedersen O, Sørensen TIA, Astrup A, Gjesing AAP, Pavo I, Wood AR, Ruetten H, Jones AG, Koopman ADM, Cederberg H, Rutters F, Ridderstrale M, Laakso M, McCarthy MI, Frayling TM, Ferrannini E, Franks PW, Pearson ER, Mari A, Hansen T, and Walker M (*J Clin Endocrinol Metab*. 2020; doi: 10.1210/clinem/dgaa653), the following transcriptional error occurred in the Advance Article version of the published paper: “Torben Hansen’s name was misspelled in the author list.”

The spelling of Torben Hansen’s name has been corrected in the final published version.

Doi: 10.1210/clinem/dgaa653

